# Appearance frequency modulated gene set enrichment testing

**DOI:** 10.1186/1471-2105-12-81

**Published:** 2011-03-20

**Authors:** Jun Ma, Maureen A Sartor, HV Jagadish

**Affiliations:** 1 Department of EECS, University of Michigan, Ann Arbor, MI, USA; 2 Center for Computational Medicine and Biology, University of Michigan, Ann Arbor, MI, USA

## Abstract

**Background:**

Gene set enrichment testing has helped bridge the gap from an individual gene to a systems biology interpretation of microarray data. Although gene sets are defined a priori based on biological knowledge, current methods for gene set enrichment testing treat all genes equal. It is well-known that some genes, such as those responsible for housekeeping functions, appear in many pathways, whereas other genes are more specialized and play a unique role in a single pathway. Drawing inspiration from the field of information retrieval, we have developed and present here an approach to incorporate gene appearance frequency (in KEGG pathways) into two current methods, Gene Set Enrichment Analysis (GSEA) and logistic regression-based LRpath framework, to generate more reproducible and biologically meaningful results.

**Results:**

Two breast cancer microarray datasets were analyzed to identify gene sets differentially expressed between histological grade 1 and 3 breast cancer. The correlation of Normalized Enrichment Scores (NES) between gene sets, generated by the original GSEA and GSEA with the appearance frequency of genes incorporated (GSEA-AF), was compared. GSEA-AF resulted in higher correlation between experiments and more overlapping top gene sets. Several cancer related gene sets achieved higher NES in GSEA-AF as well. The same datasets were also analyzed by LRpath and LRpath with the appearance frequency of genes incorporated (LRpath-AF). Two well-studied lung cancer datasets were also analyzed in the same manner to demonstrate the validity of the method, and similar results were obtained.

**Conclusions:**

We introduce an alternative way to integrate KEGG PATHWAY information into gene set enrichment testing. The performance of GSEA and LRpath can be enhanced with the integration of appearance frequency of genes. We conclude that, generally, gene set analysis methods with the integration of information from KEGG PATHWAY performs better both statistically and biologically.

## Background

Traditional microarray data analysis mainly focuses on identifying individual genes whose expression levels are associated with a certain phenotype [1]. However, to generate a biologically meaningful hypothesis based on a handful of genes is often challenging. To overcome the limitations of individual gene level analysis, gene set enrichment analysis [2] was introduced and is now widely used as a strategy for gene expression analysis over pathway knowledge. Gene set enrichment analysis using "cutoff-free" expression data integrated with prior biology knowledge generates consistent and biologically relevant results. Several gene set analysis strategies have been developed, such as GSEA [3], SAM-GS [4], LRpath [5], GAGE [6], Random Set Scoring [7], etc. Based on prior biological knowledge, such as chromosomal location, Gene Ontology term assignment, publicly available databases, conserved regulatory motif in the promoter region, etc., each of these approaches defines sets of genes that are considered to be involved in the same underlying biological process. Typically, all genes in a gene set are treated equally in subsequent analysis. However, genes involved in multiple pathways may not be as distinctive as genes specific to one pathway, and therefore may be less informative from a biological perspective. For example, specific receptors interacting with a ligand are more characteristic of a ligand-receptor pathway than signal trasduction kinases, which could play different roles in several pathways. From a gene set analysis point of view, if most of the significant differentially expressed genes in a particular gene set function in multiple pathways, the corresponding gene set might be enriched, but this result could also be a false positive result. Placing excessive concentration on differentially expressed genes with multiple roles could easily disguise the discovery of genes with less significant differential expression but higher specificity.

A similar problem has been studied extensively in the field of Information Retrieval. Given a set of search terms entered by the user, the system task is to find the most relevant documents. In evaluating the relevance of a document, the occurrence of the search terms in the document is considered. If all search terms are treated equally, it has been observed that commonly occurring terms, which are actually less useful in identifying relevant documents, swamp out the more specific search terms that occur less frequently. To address this problem, it is standard practice to weight search terms by their inverse document frequency (IDF) defined for term × as the reciprocal of (the logarithm of) the number of documents (in the repository) in which the search term occurs.

Among the many different ways to define gene sets, public databases are considered to be among the most informative, since these pathways are manually curated by experts in the area and supported by experimental evidence. KEGG (Kyoto Encyclopedia of Genes and Genomes) PATHWAY [8] is a collection of manually drawn pathway maps representing molecular interaction and reaction networks for Metabolism, Genetic Information Processing, Environmental Information Processing, Cellular Processes, and Human Diseases. As part of a systems biology approach, KEGG can be viewed as a virtual biological system containing various types of information essential for the recreation of an organism. In short, KEGG PATHWAY integrates all the molecular interaction knowledge from wet lab experiments into one database.

If we took all the pathways in KEGG as a virtual biological system, the appearance frequency of each gene could be a reliable indicator for the specificity of each gene to a pathway. Low appearance frequency genes are more specific to a pathway; if these genes show significant differential expression, then there is a good chance the corresponding pathway is activated. High appearance frequency genes function in multiple pathways; the significant differential expression of these genes could affect a number of pathways, and they are therefore less likely to lead to the identification of any particular pathway. It is also possible to observe several high appearance frequency genes cause the enrichment of truly affected pathway, while other pathways containing the same set of high frequency genes are indirectly enriched solely due to the overlapping genes. These indirectly enriched pathways interfere with the identification of the true positive pathway.

Motivated by these ideas, we propose a novel approach to characterize genes using information provided by KEGG. We propose a weighting strategy based on the appearance frequency of each gene in KEGG PATHWAY maps and incorporate it into gene set analysis methods. We applied the weights to the analysis of two independent breast cancer datasets and two independent lung cancer datasets. The results with our weights were compared with results using the original GSEA, and showed an increase in consistency between datasets. The weighting method was also incorporated into a novel logistic regression based gene set analysis method, LRpath. The combination of LRpath and our weighting strategy provided more reproducible and biologically meaningful results than using the original LRpath alone. Detailed information can be found at http://eecs.umich.edu/db/think/.

## Results and discussion

### The distribution of appearance frequency of genes in KEGG PATHWAY

**Figure 1:**
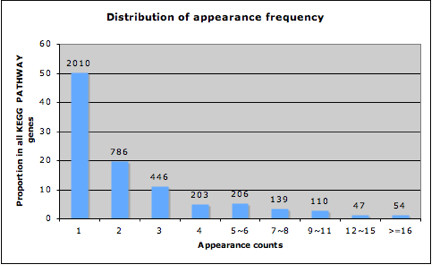
**The distribution of appearance frequency of genes in KEGG PATHWAY database**. The number under the × axis indicates the appearance frequency of each gene in KEGG PATHWAY. The y axis shows the proportion of genes with indicated appearance frequency out of all KEGG PATHWAY genes. The total number of genes at each appearance count is indicated.

We begin with an analysis of the KEGG PATHWAY database to confirm our assumptions regarding the varying appearance frequency of genes and to explore the biological basis for the observed variance. Figure 1 shows the distribution of appearance frequency of genes within KEGG PATHWAY. Half of the genes appear only once in a specific pathway. These genes are evenly distributed among all KEGG PATHWAY gene sets, without significant enrichment in any particular gene set. A decreasing proportion of genes have an increasing frequency of appearance. Less than two percent of KEGG PATHWAY genes appear more than sixteen times. This figure reflects the underlying biology of signaling pathways and the property of gene occurrence within them. If we take a closer look at the group of genes with high appearance frequency (> = 16 counts), the analysis result generated by the functional annotation tool DAVID Bioinformatics resources [9] shows that the most enriched molecular function GO term in this group is phosphotransferase activity, also known as kinase activity. A kinase is a type of enzyme that functions in phosphorylation, which is used to modify the activity of proteins, and subsequently transmit signals. MAP Kinases are known to regulate various cellular activities, especially those involved in signal transduction. An extraordinarily diverse group of cellular functions, including cell growth, proliferation, differentiation, motility, survival and intracellular trafficking, have been related to the ability of PI 3-kinases to activate Akt kinases. Mitogen-activated protein (MAP) kinase, Phosphoinositide 3-kinase, AKT family kinase and their relational proteins are all among genes with top appearance frequency.

**Figure 2:**
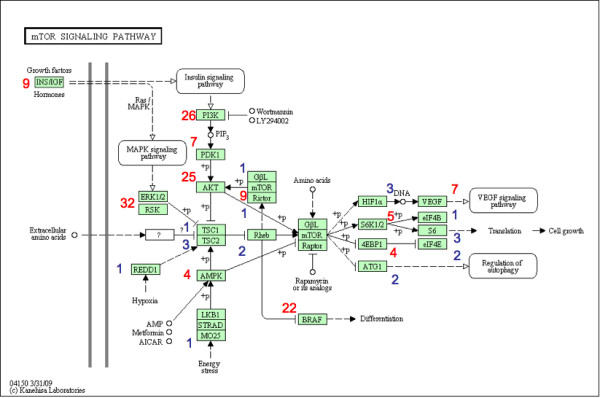
**Appearance frequencies of genes in the mTOR signaling pathway**. The appearance frequency of each gene in the mTOR signaling pathway is listed adjacent to the corresponding node in the map. Red color represents high appearance. Blue color represents low appearance. Notice that kinase genes (AKT) tend to have higher frequency compare with mTOR pathway specific genes (Raptor and Gβl).

Figure 2 shows genes involved in the mTOR signaling pathway (pathway map taken from KEGG PATHWAY, http://www.genome.jp/kegg/pathway/hsa/hsa04150.html) and their corresponding appearance frequency in KEGG PATHWAY. Current research reveals that the mTOR pathway integrates the input from multiple upstream stimuli, including environment energy stress, insulin, growth factors and mitogen [10]. In the lower part of the map, CAB39 (MO25 as shown in the map) enhances the formation of STK11/STRAD complexes. This complex is important for maintaining energy homeostasis and appears only in the mTOR signaling pathway. On the left side are Insulin and Insulin-like growth factor 1, which are involved in several cancer related pathways. The extracellular signals are transducted via the PI3K and MAPK signaling pathway. Both of them are high appearance frequency signal transduction kinases. The mTOR complex is composed of mTOR, regulatory associated protein of mTOR (Raptor) and G-protein β-like protein (GβL). Although mTOR appears in other pathways, Raptor and GβL exclusively appear in the mTOR signaling pathway. The direct downstream targets of the mTOR complex are S6K1 and 4EBP1. Activated S6K1 can in turn initiate protein synthesis and translational machinery. Activated 4EBP1 releases translation initiation factor eIF4E to perform its function. Since S6K1 and 4EBP1 are always under the control of mTOR, they have about the same appearance frequency as mTOR. The indirect target of mTOR complex, ATG1, only appears in the autophagy signaling pathway.

If we have a list of differentially expressed genes, and most of the top genes are signal trasduction kinases, such as MAPK, PI3K and AKT, we can't be sure whether the whole pathway is activated, since these genes are not specific to the mTOR signaling pathway. If S6K1 and 4EBP1 show significant changes, then there is a good chance that mTOR is also activated. If mTOR pathway specific protein Raptor and GβL are among the top genes, alone with the environmental sensing STK11/STRAD complex, then we can confidently conclude that the mTOR signaling pathway is related to the phenotype. Most other pathways in KEGG follow a similar pattern. The above example illustrates that when we interpret microarray results in terms of affected pathways, if we could put higher weight on pathway specific genes while putting less weight on genes with multiple roles, then, with the integration of this new information, the updated ranked list of significantly involved genes could become more biologically meaningful.

### Application of GSEA-AF

GSEA [3] is currently a widely used approach to gene set enrichment testing. It uses a weighted version of the Kolmogorov-Smirnov statistic to measure the degree of differential gene expression in gene sets. We decided to integrate the appearance frequency of genes in KEGG PATHWAY into GSEA.

In GSEA, genes are first ranked by signal to noise ratio. A "running sum" statistic is calculated for each gene set, based on the ranks of members in the set, relative to those of non-members. An enrichment score (ES) is defined to be the maximum of the running sum across all genes, which corresponds to a weighted Kolomogorov-Smirnov statistic. The weight places more importance on the top and bottom of the ranked list. When a gene set contains a large number of highly ranked genes, a high ES is achieved. A normalized enrichment score (NES) is calculated for each gene set based on the size of the set. NES is also used as the ranking metric to show the final result of GSEA. For each gene set, a permutation-based false discovery rate (FDR) on NES is calculated and used to identify the significance of enrichment across experiments. The FDR is adjusted for gene set size and multiple hypothesis testing. To represent biologically relevant correlation with the phenotype, the magnitude of the increment can be weighted according to each gene's correlation with the phenotype. The equation for the calculation of ES for a gene set *S *is given below, in terms of two running sums, P_hit _and P_miss_, both computed up to rank *i *in the ranked list of genes:

where r_j _is the correlation of gene expression of gene *g_j _*with the phenotype of interest, N is the total number of genes in the data set, and N_H _is the number of genes in one gene set.

The ES is the maximum deviation from zero of P_hit_-P_miss_. In the original implementation of GSEA, the running-sum statistics used equal weights at every step (exponent *p *= 0), which yielded high scores for sets clustered near the middle of the ranked list [3]. Subramanian et al addressed this issue by weighting the steps according to each gene's correlation with a phenotype, which corresponded to take *p *= 1. The exponent *p *can be adjusted to control the magnitude of each step. When *p *= 0, ES(S) reduces to the standard Kolmogorov-Smirnov statistic, when *p *= 1, the genes in one gene set are weighted by their correlation with phenotype normalized by the sum correlation over all genes in the set. We adjusted the value of the exponent *p *according to the appearance frequency of each gene. Since the correlation r_j _is 0[1], genes with high appearance frequency should receive an exponent *p *greater than 1 to reduce the magnitude of its effects. On the other hand, genes with low appearance frequency should receive an exponent *p *smaller than 1 to increase its effect. In this way, if there is a large fraction of pathway specific genes in a certain gene set ranked near the top or bottom of the list, the corresponding gene set will have a greater ES compared with the original GSEA. The appropriate value of *p *is determined based on a classical information retrieval term weighting method as described in the methods. In the following sections, we call the new model with the integration of appearance frequency into GSEA as GSEA-AF.

To demonstrate the validity of the new weighting method, we also devised two additional variants of GSEA as "controls": one based on a random appearance frequency, and another on inverse frequency. For the former, the genes are randomized while the proportion of genes with each appearance frequency is kept comparable to the original distribution. RF (Random Frequency) is used to indicate the resulting method. As another control, we generated an inverse appearance frequency, as described in the methods, for each gene in KEGG pathways and indicate this method as IW (Inverse Weight).

**Figure 3:**
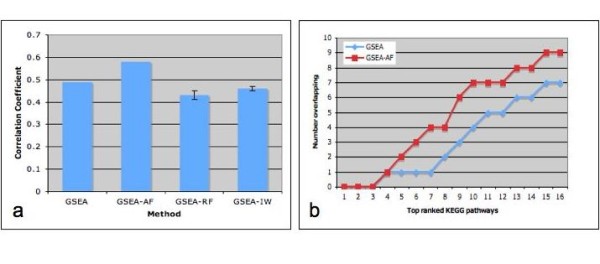
**Comparison of GSEA based methods on breast cancer datasets**. **a**. The Pearson correlation between datasets was calculated based on the Normalized Enrichment Score (NES) of each KEGG PATHWAY gene set generated by the original GSEA or with the integration of gene appearance frequency (AF), random frequency (RF), inverse weight (IW). Error bars show the standard error of the mean. **b**. Ranked list of KEGG Pathways was generated for each method and each dataset separately. The number of overlapping KEGG Pathways was calculated between datasets for each method for increasing length of ranked list. Only pathways with FDR < = 0.05 are considered.

We applied all four techniques, GSEA, GSEA-AF, GSEA-RF, and GSEA-IW, on two independent breast cancer datasets, which were originally analyzed and compared in Sartor, et al [5]. For the first dataset, human breast carcinoma samples were extracted from patients with positive estrogen receptor (ER), among which 29 samples are histological grade 1 and 12 samples are histological grade 3. In the second study, samples were taken from patients with similar condition, where 39 samples are identified as histological tumor grade 1 and 28 samples are histological tumor grade 3. Both datasets were downloaded from NCBI Gene Expression Omnibus public repository. Genes differentially expressed between histological grade 1 and 3 samples were analyzed for enrichment of KEGG pathways. As a standard to evaluate a method's performance, if there is high consistency between results obtained from the two independent yet biologically similar datasets, then the method is more reliable. Since NES is the ranking metric in GSEA, concordance is measured by the pearson correlation coefficient using the resulting NES values for each dataset as input. Figure 3a shows the consistency between the two datasets as measured by GSEA, GSEA-AF, GSEA-RF and GSEA-IW. GSEA-AF gave the highest consistency among the methods. The consistency decreased with the application of random frequency and inverse weight. Figure 3b examines the number of overlapping KEGG pathways in the top ranked list of GSEA and GSEA-AF, considering only the KEGG pathways with FDR < = 0.05. GSEA-AF identified more overlapping KEGG pathways than GSEA. Examining the overlapping gene sets with FDR < = 0.05 in the ranked list generated by GSEA and GSEA-AF (Table 1), we see that there are more overlapping gene sets discovered by GSEA-AF. More specifically, one more breast cancer related gene set was identified by GSEA-AF. In addition to cell cycle, which has already been associated with the differentiation of breast cancer state [11], it was reported that depressed expression levels of oxidative phosphorylation were observed in human breast cancer cells [12]. We also checked the improvement of analysis results in each dataset separately. Table 2 lists the NES and q-value of several cancer related gene sets generated by GSEA and GSEA-AF from the Miller et al study [13]. Most of the top gene sets received higher NES in GSEA-AF. Several metabolism pathways and non-cancer related pathways, such as Huntington's disease and type II diabetes mellitus, were moved out of the top of the ranked list.

**Table 1:** Comparison of overlapping gene sets generated by GSEA and GSEA-AF

Methods	Overlap Gene Sets (FDR < 0.05)	Cancer Related Gene Sets	Name of Gene Sets
GSEA	7	3	Proteasome, Cell cycle, Biosynthesis of steroids

GSEA-AF	9	4	Proteasome, Cell cycle, Biosynthesis of steroids, Oxidative phosphorylation

The ranked list generated by GSEA was ranked by NES. Only gene sets with q-value less than 0.05 were considered.

**Table 2:** Comparison of individual gene sets generated by GSEA and GSEA-AF

KEGG Pathway	GSEA		GSEA-AF	
	
	NES	FDR(q-value)	NES	FDR(q-value)
Cell-cycle	2.038	0.0	1.943	0.0

Oxidative phosphorylation	1.974	0.0	2.014	0.0

Biosynthesis of steroids	1.77	0.03	1.812	0.02

Proteasome	1.544	0.05	1.707	0.04

P53 signaling Pathway	1.62	0.08	1.667	0.05

Pancreatic cancer	1.385	0.21	1.612	0.08

### Application of LRpath-AF

Several gene set analysis strategies have been proposed in the literature in addition to GSEA. Following the same logic, the appearance frequency of genes in KEGG PATHWAY can be applied to these strategies as well. As an example, we modified the logistic regression-based method, LRpath, to integrate this information. The basic concept of LRpath is that if the odds of a gene belonging to a pre-defined gene set increases as the significance of differential expression increases, then this gene set is enriched. In the original LRpath, each gene is assigned a statistical significance in terms of its P-value using a test for differential expression chosen by the user. For each gene set, genes within the gene set are defined as 1, while all other genes are 0. If π is the proportion of genes belonging to the gene set at a specified significance level, then π/(1-π) are the corresponding odds that a gene with significance × is a member of this particular gene set. Logistic regression is used to model the log-odds of a gene belonging to the specific gene set as a linear function of the statistical significance -log(P-value). Whether a gene set is enriched or depleted with deferentially expressed genes is tested using the slope parameter in the logistic regression equation.

The P-values of differential expression for genes, generated by the standard t-test, were transformed to -log(P-value)s and used as input for LRpath. In addition to the significance of each gene, these values could also contain information about appearance frequencies. We integrated this information into the LRpath input as described in the methods section.

**Figure 4:**
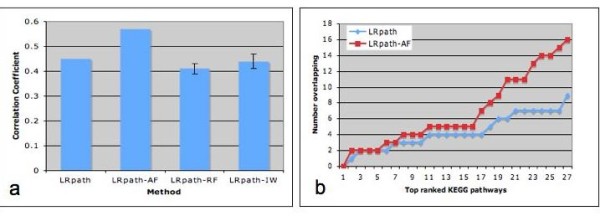
**Comparison of LRpath based methods on breast cancer datasets**. **a**. The pearson correlation between datasets was calculated based on the significance of each KEGG PATHWAY gene set generated by the original LRpath or with the integration of gene appearance frequency (AF), random frequency (RF), or inverse weight (IW). Error bars show the standard error of the mean. **b**. Ranked list of KEGG Pathways was generated for each method and each dataset separately. The number of overlapping KEGG Pathways was calculated between datasets for each method for increasing length of ranked list. Only the enriched KEGG pathways are considered (odds ratio > 1).

The same breast cancer datasets were used to compare the results of LRpath and LRpath-AF. Again the degree of correlation in significance of KEGG PATHWAYs between the two datasets was taken as the standard to measure the performance of the method. As shown in Figure 4a, results from LRpath-AF had higher correlation than LRpath. When random appearance frequency was used as input, there was no increase in correlation. The correlation of LRpath-IW was not significantly different than the correlation given by LRpath, and was significantly lower than the correlation given by LRpath-AF. Among all the methods we have tested, LRpath generally performed better than GSEA. LRpath-AF has the highest correlation. Figure 4b examines the number of overlapping KEGG pathways in the top ranked list of LRpath and LRpath-AF, considering only the enriched KEGG pathways (odds ratio > 1). LRpath-AF identified more overlapping KEGG pathways than LRpath. In addition to cell cycle and Proteasome gene sets, which were identified by GSEA, a well-known cancer related pathway, p53 signaling pathway, was discovered as significant by LRpath-AF.

### Comparing results from two studies of lung cancer

To further demonstrate the advantage of applying appearance frequency of genes in gene set analysis, we applied the same methods as above on two lung cancer datasets, which have been analyzed by the original GSEA method [3] and several other methods [6]. These two datasets were generated independently by research groups in Boston (62 samples) and Michigan (86 samples). Gene expression profiles in tumor samples from patients with lung adenocarcinomas were obtained by microarray. Based on clinical outcomes, the samples were classified as having a "good" or "poor" outcome. The patients with "good" clinical outcome were defined as control, and differentially expressed genes associated with "poor" outcome were identified. The individual gene level analysis did not show any significant overlap between datasets. The application of GSEA was able to detect more similarities and provided biological insights through the identification of overlapping gene sets.

**Figure 5:**
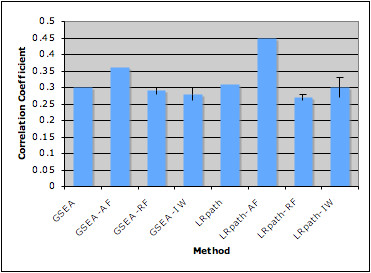
**Correlation between lung cancer datasets across methods**. The pearson correlation between datasets was calculated based on results generated by GSEA, GSEA-AF, GSEA-RF, GSEA-IW, LRpath, LRpath-AF, LRpath-RF, LRpath-IW. Error bars show the standard error of the mean.

We compared the results of GSEA, GSEA-AF, GSEA-RF, GSEA-IW, LRpath, LRpath-AF, LRpath-RF, and LRpath-IW on these two datasets following the same procedure as described above. As shown in Figure 5, incorporating the appearance frequency of genes reveals greater consistency between datasets. The correlations of results are not affected by the integration of random frequency. Among all methods, LRpath-AF resulted in the highest correlation. The top ranked list generated by LRpath-AF also shows more overlapping enriched gene sets. Compared with the original LRpath, more cancer disease gene sets are among the top using LRpath-AF, including Non-small cell lung cancer. Several other cancer related signaling pathways and cellular processes are significantly enriched as well, such as VEGF signaling pathway, which is essential for the sustained angiogenesis in tumor growth [14].

## Conclusions

Current statistical approaches are able to identify significantly differentially expressed genes individually, but knowing a list of significant genes is not sufficient to make conclusions about the underlying biological processes. Development of gene set analysis methods has made the interpretation of microarray results at a systems biology level much easier. Instead of focusing on individual genes, researchers can focus on gene sets, which give more reproducible and interpretable results. Without requiring an arbitrary cutoff of significance, the changes of all genes in an experiment can be considered.

Although the construction of gene sets is based on biological knowledge, most current methods fail to take full advantage of the available resources. Recently, there have been efforts to bring more pathway-specific information into the analysis of microarray data. Draghici et al [15] reported a novel impact analysis method, in which they tried to capture a number of additional factors that may affect the analysis results. Additional information from KEGG PATHWAY, such as the position of the differentially expressed genes in the pathways, the topology of the pathway that describes gene relations and the types of interactions among them, were taken into account in the analysis of differentially expressed genes. The latest publication from the same group further refined the original model to reduce the false positive rate for short lists of differentially expressed genes and to make the model less sensitive to noise in the expression data [16]. The impact analysis is a good combination of a traditional statistical approach with biology knowledge. However there is no discussion of appearance frequency.

In this paper, we provided the motivation for and proposed a strategy to integrate more biologically meaningful information into gene set analysis. First, the appearance frequency of each gene in KEGG pathways was determined. Further analysis showed that the appearance frequency of genes has potential relations to the property of the gene. Greater appearance frequency indicates less specificity to a particular gene set, and therefore more likely to create a false positive result. Fewer appearances indicate the function of the gene is important to a particular biological process. The significant changes of low appearance genes are more likely to be highly related to the perturbation of the corresponding pathway. We adopted a classical approach in information retrieval to determine the weight to be used for adjustment of the methods. The performance of each method was assessed using two independent breast cancer data sets and two independent lung cancer datasets. The observed concordance of the results was improved with the integration of appearance frequency of genes. Compared with original GSEA and LRpath, the updated versions give more reproducible and biologically meaningful results. The successful integration of the appearance frequency of genes into GSEA and LRpath also suggests the potential to apply this information to other gene set analysis methods.

## Methods

### Determination of the exponent value p in GSEA-AF

To determine the appropriate value for the exponent *p*, we adopt a classical information retrieval term weighting method [17]. The importance of a term in a given document can be estimated by multiplying the raw term frequency (TF) of the term in a document by the term's inverse document frequency (IDF) weight. The importance increases proportionally to the number of times a word appears in the document but is offset by the frequency of the word in a collection. This method was easily transformed for our purpose. Each pathway map is composed of a group of genes, which is the analog of a document and the words in it. Since most genes only appear once in a given pathway map, the term frequency doesn't provide further useful information in our case. There are 195 KEGG pathway maps in KEGG database, which could be viewed as a collection of documents. The inverse term frequency is a measure of general importance of the term. IDF is obtained by dividing the number of all documents by the number of documents containing the term, and then taking the logarithm. In our case, the number of documents containing the term is the appearance frequency of genes across all KEGG PATHWAY gene sets. In order to make genes with low appearance having a higher weight, the exponent *p *needed to be adjusted to less than 1. We used the average of IDF of all genes as a criterion to determine the level of appearance frequency. The exponent *p *is determined as below:

where *idf_i _*defined as *idf_i _*= log(195/*f_i_*), *f_i _*is gene appearance frequency, i = 1,..., n.

This resulted in the exponent *p *ranging from 0.73 to 2. When the appearance frequency is 4, the exponent *p *approximately equals 1. Low appearance frequency genes get an exponent *p *less than 1, while high appearance genes get an exponent *p *greater than 1, which leads to a smaller weight. In additional validations, using more (or less) extreme ranges of the exponent *p *did not continue to improve results, as assessed by correlation between the breast cancer datasets and lung cancer datasets (additional file 1).

### Generation of inverse appearance frequency

The purpose of using inverse appearance frequency and random appearance frequency is to make sure the positive effect of appearance frequency on GSEA and LRpath is specific, rather than an artifact of the method in general. In GSEA-RF, appearance frequencies were assigned to each gene randomly, while the proportions of genes with each appearance frequency were kept comparable to the original distribution. In GSEA-IW and LRpath-IW, higher weights were put on genes that are involved in multiple pathways.

Our method for running the IW tests preserves the true distribution of numbers of pathways to which genes belong. We generated an inverse appearance frequency for each gene in KEGG pathways based on their actual appearance frequency. For example if one gene has appearance frequency of 1, a random appearance frequency between 20-30 is taken as its inverse appearance frequency. If one gene has appearance frequency greater than 20, the inverse appearance frequency of this gene is set to 1. This way the true distribution of appearance frequency of all genes in KEGG pathways are approximately preserved, and the adjustment on the exponent *p *in IW tests is also closer to the scale used for GSEA-AF and LRpath-AF. Ten sets of inverse appearance frequency were generated and used as *f_i _*in equation for determine exponent p in IW tests. The mean and the standard error of the mean of the correlations in IW tests were calculated.

### Application of appearance frequency in LRpath

Appearance frequency can be applied to different significance measures in LRpath. In the original LRpath, the statistical significance of each gene is represented by its P-value. We applied the exponent *p *on the -log(P-value), since LRpath performs logistic regression on this P-value transformation and it preserves the information about the relative significance levels in the P-values. After the -log(P-value) transformation, genes with lower appearance frequency should have greater -log(P-value) to enhance their significance, while genes with higher appearance frequency should move down in significance, corresponding to a lower -log(P-value). We again adopted the idea of TF-IDF described above to determine the parameter used for adjusting the -log(P-values). Finally, the appearance frequency adjusted -log(P-values) were passed into LRpath for analysis.

## Authors' contributions

The central idea was conceived in discussions between all three authors. The code implementation and evaluation experiments were conducted by JM under the direction of the other authors. The initial manuscript draft was also prepared by JM, and then substantially edited by the other authors. All authors read and approved the final manuscript.

## Supplementary Material

Additional file 1 **Supplementary_material_AF_paper**. This file describes the test results on using extreme range of exponent *p*. Click here for file
