# Supplementary material for: Appearance frequency modulated gene set enrichment testing
Source: BMC Bioinformatics. 2011 Mar 20;12:81. doi: 10.1186/1471-2105-12-81 (PMC3213687; doi:10.1186/1471-2105-12-81)

Using extreme ranges of the exponent *p* doesn’t continue to improve correlation as shown in Figure S1 and Figure S2. Increasing exponents were put on *p* to observe the effect on the output of GSEA-AF. For each adjusted exponent value, we performed the same analysis using the same datasets as described in the text and calculated the correlation of output rank list. Figure S1 is based on the breast cancer data and Figure S2 is based on the lung cancer data.


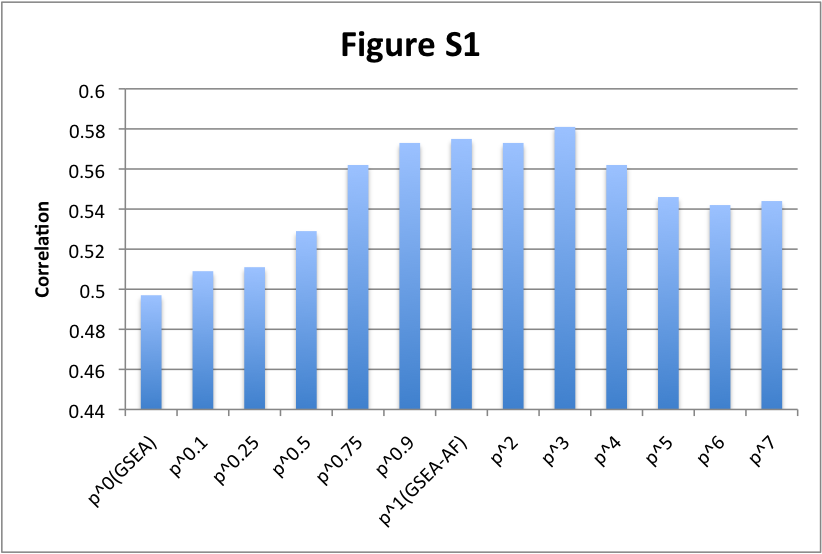


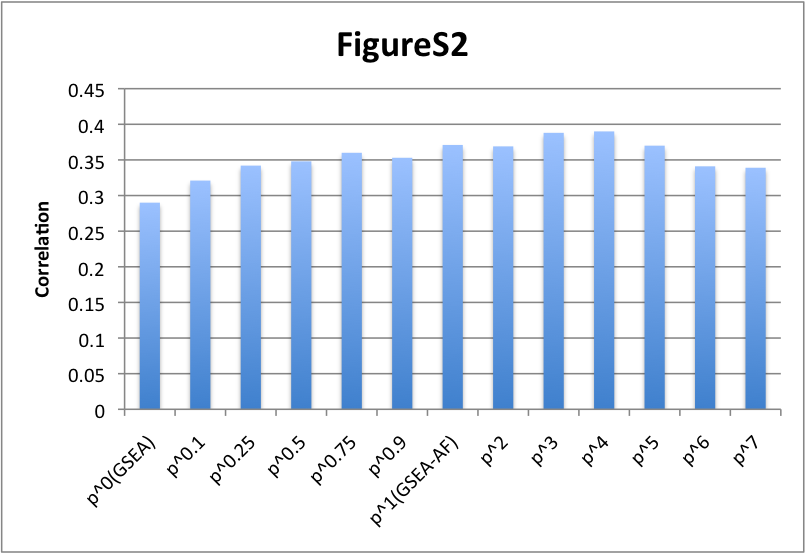

Supplement: Additional file 1 — Supplementary_material_AF_paper. This file describes the test results on using extreme range of exponent p. [file 1471-2105-12-81-S1.DOC]
